# Supporting Patient Engagement in Dementia Research via Technology During the COVID-19 Pandemic

**DOI:** 10.1093/geroni/igab046.1205

**Published:** 2021-12-17

**Authors:** Lillian Hung, Sophie Yang, Mario Gregorio, Alison Phinney

**Affiliations:** 1 University of British Columbia, Vancouver, British Columbia, Canada; 2 Vancouver, British Columbia, Canada

## Abstract

The COVID-19 pandemic brings challenges to patient partnerships in research. In-person research meetings with patient partners were prohibited. In this presentation, we outline specific issues we encountered in a patient-led dementia research project, which involved a literature review study and gathering community stakeholders to identify the top 10 local priorities in the development of a dementia-friendly community. We will describe how we found shared solutions to complete the project. In response to COVID, computers and training were provided for patient partners to maintain team connection, plan project activities, conduct team analysis, and host a community workshop in the lockdown time. The drastic shift to virtual research methods created barriers and opportunities for co-research with older people with dementia. Virtual meetings can generate inequities for those who do not have a computer and knowledge in videoconferencing. Practical strategies to overcome barriers to using virtual technologies will be explored.

